# Antioxidant Properties of Brazilian Tropical Fruits by Correlation between Different Assays

**DOI:** 10.1155/2013/132759

**Published:** 2013-09-10

**Authors:** Elena Gregoris, Giuseppina Pace Pereira Lima, Sabrina Fabris, Mariangela Bertelle, Michela Sicari, Roberto Stevanato

**Affiliations:** ^1^Department of Molecular Sciences and Nanosystems, University Ca' Foscari of Venice, Dorsoduro 2137, 30123 Venice, Italy; ^2^Institute of Biosciences, UNESP University, Campus of Botucatu, CP 510, 18618-000 Botucatu, SA, Brazil

## Abstract

Four different assays (the Folin-Ciocalteu, DPPH, enzymatic method, and inhibitory activity on lipid peroxidation) based on radically different physicochemical principles and normally used to determine the antioxidant activity of food have been confronted and utilized to investigate the antioxidant activity of fruits originated from Brazil, with particular attention to more exotic and less-studied species (jurubeba, *Solanum paniculatum*; pequi, *Caryocar brasiliense*; pitaya, *Hylocereus undatus*; siriguela, *Spondias purpurea*; umbu, *Spondias tuberosa*) in order to (i) verify the correlations between results obtained by the different assays, with the final purpose to obtain more reliable results avoiding possible measuring-method linked mistakes and (ii) individuate the more active fruit species. As expected, the different methods give different responses, depending on the specific assay reaction. Anyhow all results indicate high antioxidant properties for siriguela and jurubeba and poor values for pitaya, umbu, and pequi. Considering that no marked difference of ascorbic acid content has been detected among the different fruits, experimental data suggest that antioxidant activities of the investigated Brazilian fruits are poorly correlated with this molecule, principally depending on their total polyphenolic content.

## 1. Introduction

It is known that the consumption of fruit and vegetable reduces the incidence of cardiovascular and cerebrovascular diseases, stroke, cancer, and ageing related disorders [1–3]. This effect is attributed to the presence in fruit and vegetables of antioxidants able to preserve the correct balance oxidants/antioxidants, in which upset due to an overproduction of oxygen reactive species (ROS) can lead to the so-called “oxidative stress” [4–6].

Substantial damages have been observed when ROS interact with DNA, membrane lipids, and proteins [7–10]. ROS are involved in the carcinogenic stages of initiation, promotion, and progression [11]; they play an important role in the development of cardiovascular diseases such as ischemic injury, arteriosclerosis, hypertension, cardiomyopathies, congenital heart diseases, and stroke; they may be a causal factor of neurological disorders such as Alzheimer's and Parkinson's diseases [12]. 

Antioxidant substances represent one of the most important defense mechanisms against free radicals, but the only endogenous antioxidant molecules cannot be effective enough to counteract the injuries caused by ROS, particularly in the current times, where lifestyles based on smoke, drugs, alcohol, unbalanced diet, pollution, incorrect exposure to solar radiation, and so forth can facilitate free radicals formation. For this reason increasing the intake of dietary antioxidant is of great importance to enjoy good health, as evidenced by studies on food characterized by high antioxidants content [13].

Unfortunately, no reliable biomarker of antioxidant activity is available up to now [14, 15] because ROS injuries are mediated by different radical and nonradical species which show different physicochemical characteristics and reaction mechanism affecting reactivity, selectivity, partition in aqueous and lipid phase, and so forth [16]. In literature many experimental methods are reported to determine a generic antioxidant activity of a compound, but results obtained by different investigations are frequently contradictory [17]. 

The aim of this work is to compare of the results obtained by four different methods usually employed to measure antioxidant properties, that is, reducing capacity by the Folin-Ciocalteu assay, radical scavenging ability towards 2,2′-diphenyl-1-picrylhydrazyl (DPPH method), inhibitory ability on peroxidation of linoleic acid (LA), and total phenolic determination by the enzymatic method [18] in order to (i) verify possible correlations between the results obtained and (ii) obtain more reliable results avoiding possible measuring-method linked mistakes. 

These assays were applied to a series of Brazil fruits, with particular interest in the more exotic and less studied species. In fact, information on the nutritional values of the most exotic species of tropical fruits are limited: some studies [19–22] provide evidence for the high antioxidant capacity and significant amounts of flavonoids and vitamin C for the most common Brazilian fruits as mango [23], starfruit [19], and avocado [24], but no data are reported for more exotic fruits, like pitaya, jurubeba, siriguela, and pequi, some of which native peoples utilize in popular medicine.

The results obtained by these measurements were compared with each other and with those obtained by Italian soft fruits known for their antioxidant activity [25, 26]. Furthermore, to discriminate possible interferences due to ascorbic acid and anthocyanins, the content of these reducing molecules in all fruits was also carried out. 

Similitude and differences were discussed on the light of the chemical characteristics of the assay reactions.

## 2. Materials and Methods

### 2.1. Chemicals

 All chemicals, of the highest available quality, were obtained from Sigma Chemical Co. (St. Louis, USA); ABIP (2,2′-azobis[2′-(2-imidazolin-2-yl)propane] dihydrochloride) was obtained from Wako Chemicals (Germany). The aqueous solutions were prepared with quality milli-Q water. Each experiment was in triplicate.

### 2.2. UV-VIS and Electrochemical Measurements

 Spectrophotometric measurements were recorded on a UV-VIS Shimadzu UV-1800 instrument equipped with a temperature controlled quartz cell. The measures of oxygen consumption were performed with a potentiostat Amel 559, equipped with an oxygen microelectrode (MI-730, Microelectrodes).

### 2.3. Fruits and Sample Pretreatments

Table 1 reports common and scientific names of all studied fruits. Mango, avocado, carambola, and pitaya were from Sao Paulo state, while jurubeba, umbu, graviola, pequi, siriguela, and tamarind were from tropical Brazil; soft fruits were from Italy. After cleaning with distilled water, edible fruits portions were grated and centrifuged by a Krups centrifuge under nitrogen flux to avoid the oxidation of the natural components, and the juice was immediately analysed.

### 2.4. Inhibition of Lipid Peroxidation (ILP)

 The antioxidant activity of fruits to prevent linoleic acid (LA) peroxidation was determined in sodium dodecyl sulfate (SDS) micelles. As previously reported [27], the fruit's antioxidant capacity was calculated as the juice concentration (ppm) halves the rate of oxygen consumption due to the peroxidation process, and it is expressed as inhibitory concentration IC_50_.

### 2.5. 2,2-Diphenyl-1-picrylhydrazyl (DPPH) Radical Scavenging Capacity Assay

 This method is based on the capacity of an antioxidant to scavenge the stable free radical DPPH [28]. The procedure is reported in Stevanato et al. [18]; the results are expressed as catechin equivalent concentration (CE).

### 2.6. Folin-Ciocalteu Assay and Total Phenolics Content (TPC) by Enzymatic Method

 The Folin-Ciocalteu assay and the Total Phenolic Content were determined spectrophotometrically, according to the procedures previously reported [18], and the results were expressed as catechin equivalent (CE).

### 2.7. Total Hydroxycinnamic Acid Content (HCA)

 Hydroxycinnamic acid content was determined according to Zaporozhets et al. [29]. The complex of hydroxycinnamic acids with aluminium (III) was measured at 365 nm, and caffeic acid was used as a standard; the results were expressed as milligrams/liter of caffeic acid equivalents. 

### 2.8. Total Anthocyanin Content (TAC)

 The TAC was determined according the pH-differential method [30]. Absorbance at 510 and 700 nm of juice buffered at pH 4.5 e 1.0 was calculated. The anthocyanin concentration was expressed as milligrams/liter of cyanidin-3-glucoside equivalents.

### 2.9. Total Ascorbic Acid (TAA)

 The TAA is assayed as previously described [31] with minor modifications. A 20 mM oxalic acid solution containing the sample, 0.186 mM 2,6-dichlorophenol-indophenol (DCFI), 10 mM dinitrophenylhydrazine (DNPH), and 13 mM thiourea were incubated in a boiling water bath for 15 minutes. Once cooled, an equal volume of 85% sulfuric acid was added to the solution, and the absorbance at 520 nm was measured 15 minutes later. The same procedure was repeated without the sample, and the blank value was subtracted from the absorbance of the sample. 

## 3. Results

In Table 2, where the results obtained by applying ILP, Folin, DPPH, and TPC enzymatic methods are reported, it appears that jurubeba and siriguela show very low IC_50_ values (i.e., high antioxidant activity) in the range of those found for the more active Italian soft fruits (blueberry, redcurrant, and raspberry). For the same fruits, DPPH, TPC, and Folin assays give very high values of CE, if compared with the average of other fruits, indicating an univocal high antioxidant activity of these two fruits.

**Figure 1:**
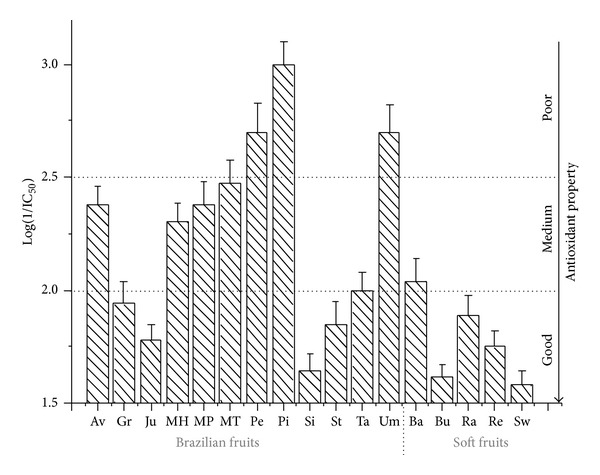
Classification of studied fruits on the base of their logarithm IC_50_ values.

On the basis of their IC_50_ values, the investigated Brazilian tropical fruits can be roughly divided into three groups characterized approximately by good, medium, and poor antioxidant properties, respectively (Figure 1): (1) fruits with log⁡(IC_50_) ≤ 2 (IC_50_ ≤ 100 ppm): graviola, jurubeba, siriguela, carambola, and tamarind; (2) fruits with 2 < log⁡(IC_50_) ≤ 2.5 (IC_50_ ranging from 100 to 316 ppm): avocado and mango; (3) fruits with log⁡(IC_50_) > 2.5 (IC_50_ > 316 ppm): pequi, umbu, and pitaya. 

**Figure 2:**
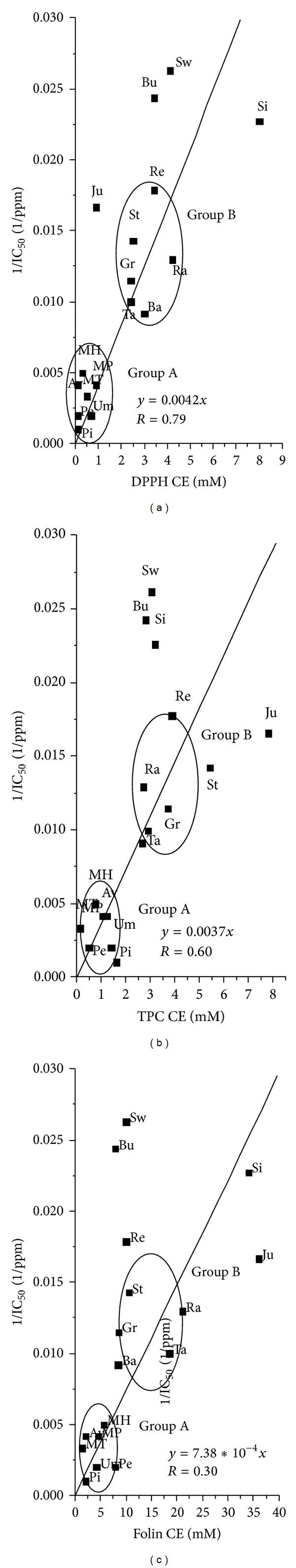
Correlation between ILP and (a) DPPH, (b) enzymatic, and (c) Folin assay.

In Figure 2, correlations between data obtained by ILP expressed as 1/IC_50_ and other adopted methods expressed as catechin equivalent amount (CE) are reported.

The comparison of the data obtained by ILP versus DPPH scavenging methods (Figure 2(a)) points out a good correlation (*R* = 0.79); in fact only few points referred to that strawberry, blueberry, jurubeba, and, in less amount, siriguela scatter from the linear relationship. 

Analogous graph created for comparison of ILP with enzymatic or the Folin methods (Figures 2(b) and 2(c)) shows less good correlations (*R* = 0.60 and 0.30, resp.), but also in this case strawberry, blueberry, jurubeba, and, in part, siriguela appear to worsen the correlation coefficient. 

TAC measurements showed the absence of anthocyanins in analyzed Brazilian fruits, while as regards the hydroxycinnamic acid content, the values of HCA equivalents obtained for the studied fruits and reported in Table 2 show a very high value of HCA_ eq_ for jurubeba.

 No correlation appears comparing TAA values with the data obtained by the other analytical methods (data are not showed).

## 4. Discussion

### 4.1. On the Assay Methods

Several methods are proposed to evaluate the antioxidant activity of molecules or food [26–28, 32–35]. Each assay measures a specific chemical or physicochemical parameter which can be correlated with the complex and in part unknown mechanisms related to ROS injury. It follows that the results obtained are partial and sometime are affected by other variables not strictly correlated to the antioxidant activity. In this work, we chose four different assays which significantly represent the main methods of measuring the antioxidant properties of a substance.

The Folin-Ciocalteu is a very aged and largely used assay, based on the absorbance changes due to the oxidation of any reduced compounds by a phosphomolybdate and phosphotungstate solution. It is a nonspecific method of measuring the reducing capacity of all the components of the sample other than polyphenols, such as ascorbate [18, 36]. In fact, to avoid an overestimated evaluation of the antioxidant capacity, laborious pretreatments of the sample are suggested [37].

TPC enzymatic method, on the contrary, being a measure of the total phenolic content of fruit due to the specificity of peroxidase-catalyzed reaction towards phenolic structures, is an indirect evaluation of the antioxidant power, which actually depends not only on the measured total phenolic content, but also on the chemical structure of each phenolic component [14]. 

DPPH method is a measure of the electronic transfer from the phenolic structure to the stable free radical DPPH, but this reaction presents the following disadvantages which can underestimate the antioxidant capacity:

- it may react slowly or be inert to many antioxidants [38];
- reaction kinetic with antioxidants appears not linear to DPPH concentrations [36];
- reaction of DPPH with some phenolic structures could not go to completion, reaching an equilibrium state, as found for eugenol [36].

By a physicochemical point of view, ILP technique appears to better reproduce the *in vivo* action of antioxidant substances against radical-induced lipid peroxidation of unsaturated fatty acids residues of biological membranes, measuring *in vitro* the slowdown, due to an antioxidant, of the oxygen consumption in linoleic acid containing SDS micelles. In this case, the influence due to the different lipophilicity of the antioxidant molecules is taken in account too. Moreover, in this work, only clear juices have been analyzed, and, as a consequence, only water soluble antioxidants have been assessed.

Anyway, in order to be certain of the data reliability and to give a wider outlook of the problems related to the definition of the antioxidant activity of foods, the same samples were studied by the above cited four analytical assays, and the results were compared to put in light possible correlations. In fact, good correlations between results obtained by different assays can guarantee the best evaluation of the antioxidant properties of a sample.

### 4.2. On the Antioxidant Characteristics of Brazilian Fruits

Siriguela, jurubeba, carambola, graviola, and tamarind show high antioxidant activity, similar to that of soft fruits [25, 26]. This result appears very important considering that for some of these fruits no information in literature is reported, in particular about their antioxidant properties [39]. Moreover, the widespread use for curative actions into local populations of some of these fruits, in particular jurubeba and siriguela, suggests further investigations for their possible nutraceutical properties.

**Figure 3:**
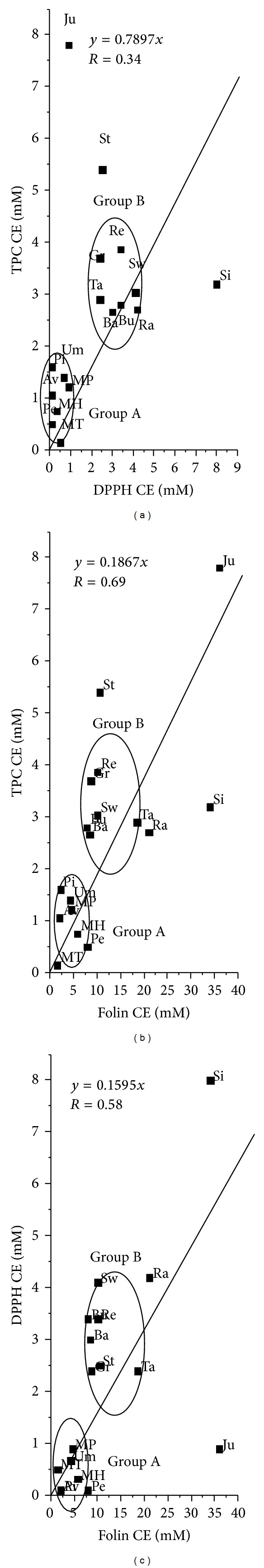
Correlation between (a) enzymatic and DPPH; (b) enzymatic and Folin; (c) DPPH and Folin assays.

With reference to the scattering from the linear correlation of the data referred to strawberry, blueberry, jurubeba, and siriguela, as it results in all three graphs of Figure 2, plots of correlation of the data obtained by DPPH, Folin, and enzymatic methods are graphed in order to verify if this deviation could be due to a limit of the ILP assay (Figure 3). Also in these cases, the data of the above-mentioned fruits appear considerably out of the correlation straight line, indicating that the chemical compounds that are responsible of the antioxidant activity are differently recorded by the different analytical methods. 

Jurubeba and siriguela are two striking examples of how different assays may assign different rankings to antioxidant molecules: as it appears in Figure 3(a), while the antioxidant activity of jurubeba is high when evaluated by the enzymatic method and low when evaluated by DPPH, in the case of siriguela the DPPH method assigns it excellent antioxidant properties which are not confirmed by the enzymatic assay. The result of the first case can be due to the high content in jurubeba of polyphenols characterized by a low tendency to undergo monoelectronic transfer to DPPH, as recently verified for different flavonoids [14]. Further investigations to clarify this contrasting behaviour are necessary in any case.

The better correlation results from the comparison of the ILP and DPPH data (Figure 2(a)). In fact, both the analytical methods are based on the redox potentials of the monoelectronic transfer, and they appear in some way as a direct measure of the radicals stopping power [28, 39] of the antioxidant substances in the fruit. Moreover, the joint data obtained by IC_50_ and DPPH experiments are particularly efficient for separating poor antioxidants from good ones: IC_50_ values that are lower than 100 ppm and/or CE values that are higher than 2 mM could be assumed as a reasonable rule for discriminating very good antioxidants. 

Even if there is a bad correlation between DPPH and enzymatic data (Figure 3(a)), most of the fruit can be roughly separated in two groups (A and B) with different degrees of antioxidant activity, suggesting the hypothesis that fruit of the same group could have quite similar compositions of antioxidant constituents or molecules which react in similar way to the analytical methods. 

**Figure 4:**
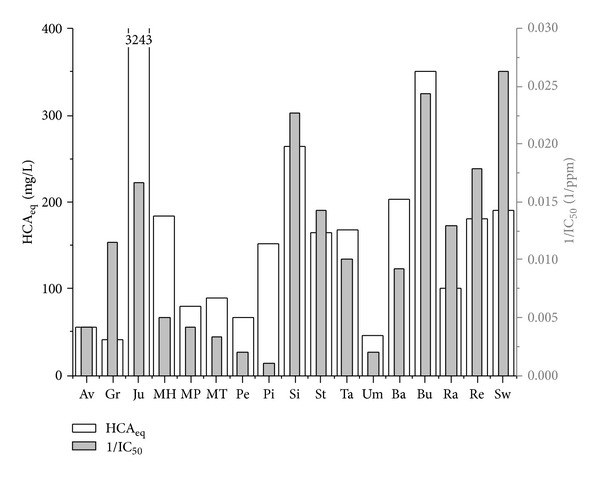
Correlation between ILP and HCA equivalents.

Anthocyanins are not contained in examined Brazilian fruits, while hydroxycinnamic acids are detected; their correlation with ILP is practically absent, as shown in Figure 4. For this reason, antioxidant property must depend on other parameters.

Table 2 indicates that, in general, Brazilian fruits have ascorbic acid content comparable to that of soft fruits: among them two varieties of mango and tamarind have meaningfully high TAA content, and umbu have the lowest one. 

No evident relationship between the antioxidant activity of fruit and the content of ascorbic acid is observed: siriguela and jurubeba have the highest antioxidant activity, but they exhibit lower values of vitamin C than mango, which is not a good antioxidant instead (Table 2). It follows that antioxidant activity of the majority of fruits is due to compounds different from vitamin C, like polyphenols, mainly flavonoids, according to results reported for other species of fruit [11, 22].

## 5. Conclusion

Brazilian fruits were used as arbitrary alimentary products to compare four different assays normally utilised to determine antioxidant activity of food.

The better correlation was found between the inhibition of lipid peroxidation and DPPH method. Both these assays are based on monoelectronic transfer, and, in our opinion, they mime, more than others, the efficacy of an antioxidant compound to prevent oxidative damage on cell membrane, despite all the limitations of the DPPH assay above reported and taking into account the laboriousness of the ILP method. From data obtained by these two methods, siriguela and jurubeba show the higher antioxidant activity. 

The antioxidant activity of the majority of the studied fruit is due to compounds different from vitamin C, like flavonoids, because no evident relationship between the antioxidant activity of fruit and the content of ascorbic acid was observed.

## Figures and Tables

**Table 1:** Selected fruits and their abbreviation.

Scientific name	Common name	Abbreviation
*Persea americana *	Avocado	Av
*Annona muricata *	Graviola	Gr
*Solanum paniculatum *	Jurubeba	Ju
*Mangifera indica *	Mango Haden	MH
*Mangifera indica *	Mango Palmer	MP
*Mangifera indica *	Mango Tommy Atkins	MT
*Caryocar brasiliense *	Pequi	Pe
*Hylocereus undatus *	Pitaya	Pi
*Spondias purpurea *	Purple mombin (siriguela)	Si
*Averrhoa carambola *	Starfruit (carambola)	St
*Tamarindus indica *	Tamarind	Ta
*Spondias tuberosa *	Umbu	Um
*Rubus ulmifolius *	Blackberry	Ba
*Vaccinium cyanococcus *	Blueberry	Bu
*Rubus idæus *	Raspberry	Ra
*Ribes rubrum *	Redcurrant	Re
*Fragaria *	Strawberry	Sw

**Table 2:** Results obtained by ILP, DPPH, TPC, Folin, HCA, and TAA assays of selected fruits.

Fruit	Abbreviation	ILP IC_50_ (ppm)	DPPH CE (mM)	TPC CE (mM)	Folin CE (mM)	HCA (mg/L)	TAA (mM)
Avocado	Av	240 ± 20	0.1 ± 0.01	1.06 ± 0.05	1.99 ± 0.04	56 ± 6	3.1 ± 0.2
Graviola	Gr	87 ± 9	2.4 ± 0.6	3.7 ± 0.5	8.6 ± 0.4	42 ± 3	4.7 ± 0.2
Jurubeba	Ju	60 ± 8	0.9 ± 0.3	7.8 ± 0.5	36 ± 2	3242 ± 20	3.4 ± 0.2
Mango Haden	MH	200 ± 20	0.31 ± 0.05	0.75 ± 0.09	5.7 ± 0.2	184 ± 12	8.6 ± 0.8
Mango Palmer	MP	240 ± 20	0.89 ± 0.01	1.21 ± 0.05	4.5 ± 0.3	80 ± 9	5 ± 1
Mango Tommy Atkins	MT	300 ± 20	0.50 ± 0.07	0.15 ± 0.03	1.4 ± 0.1	90 ± 10	3.7 ± 0.6
Pequi	Pe	500 ± 50	0.1 ± 0.01	0.5 ± 0.1	7.9 ± 0.2	66 ± 7	2.4 ± 0.3
Pitaya	Pi	1000 ± 100	0.1 ± 0.01	1.6 ± 0.2	2.1 ± 0.2	152 ± 12	2.6 ± 0.2
Siriguela	Si	44 ± 4	8 ± 1	3.2 ± 0.1	34 ± 5	264 ± 23	4.7 ± 0.3
Carambola	St	70 ± 7	2.5 ± 0.1	5.4 ± 0.4	10.5 ± 0.1	164 ± 14	4.2 ± 0.3
Tamarind	Ta	100 ± 20	2.4 ± 0.3	2.9 ± 0.1	18.5 ± 0.8	168 ± 15	7 ± 1
Umbu	Um	500 ± 30	0.67 ± 0.05	1.4 ± 0.2	4.2 ± 0.1	46. ± 3	1.5 ± 0.2
Blackberry	Ba	109 ± 6	3.0 ± 0.2	2.66 ± 0.06	8.4 ± 0.1	203 ± 19	4.4 ± 0.6
Blueberry	Bu	41 ± 7	3.4 ± 0.1	2.8 ± 0.1	7.8 ± 0.5	350 ± 25	3.0 ± 0.1
Raspberry	Ra	77 ± 9	4.2 ± 0.1	2.7 ± 0.1	21 ± 1	101 ± 9	3.7 ± 0.3
Redcurrant	Re	56 ± 2	3.4 ± 0.4	3.9 ± 0.4	10.0 ± 0.1	180 ± 12	4 ± 0.4
Strawberry	Sw	38 ± 4	4.1 ± 0.3	3.04 ± 0.09	10 ± 3	190 ± 13	6 ± 1
